# Pattern of injury mortality by age-group in children aged 0–14 years in Scotland, 2002–2006, and its implications for prevention

**DOI:** 10.1186/1471-2431-9-26

**Published:** 2009-04-07

**Authors:** Janne Pearson, David H Stone

**Affiliations:** 1 Paediatric Epidemiology and Community Health (PEACH) Unit, Faculty of Medicine, University of Glasgow, Glasgow, UK

## Abstract

**Background:**

Knowledge of the epidemiology of injuries in children is essential for the planning, implementation and evaluation of preventive measures but recent epidemiological information on injuries in children both in general and by age-group in Scotland is scarce. This study examines the recent pattern of childhood mortality from injury by age-group in Scotland and considers its implications for prevention.

**Methods:**

Routine mortality data for the period 2002–2006 were obtained from the General Register Office for Scotland and were analysed in terms of number of deaths, mean annual mortality rates per 100,000 population, leading causes of death, and causes of injury death. Mid-year population estimates were used as the denominator. Chi-square tests were used to determine statistical significance.

**Results:**

186 children aged 0–14 died from an injury in Scotland during 2002–06 (MR 4.3 per 100,000). Injuries were the leading cause of death in 1–14, 5–9 and 10–14 year-olds (causing 25%, 29% and 32% of all deaths respectively). The leading individual causes of injury death (0–14 years) were pedestrian and non-pedestrian road-traffic injuries and assault/homicide but there was variation by age-group. Assault/homicide, fire and suffocation caused most injury deaths in young children; road-traffic injuries in older ones. Collectively, intentional injuries were a bigger threat to the lives of under-15s than any single cause of unintentional injury. The mortality rate from assault/homicide was highest in infants (<1 year) and decreased with increasing age. Children aged 5–9 were significantly less likely to die from an injury than 0–4 or 10–14 year-olds (p < 0.05). Suicide was an important cause of injury mortality in 10–14 year-olds.

**Conclusion:**

Injuries continue to be a leading cause of death in childhood in Scotland. Variation in causes of injury death by age-group is important when targeting preventive efforts. In particular, the threats of assault/homicide in infants, fire in 1–4 year-olds, pedestrian injury in 5–14 year-olds, and suicide in 10–14 year-olds need urgent consideration for preventive action.

## Background

Injuries are an important cause of death in children both in the United Kingdom and worldwide. [1-3] Prevention strategies appear to have produced steep declines in unintentional injury mortality rates in children over recent years in developed countries, including in Scotland. [4] To ensure continuing success it is important to keep pace with changes in levels of risk and to highlight emergent ones. There is little recent information on the epidemiology of deaths from injuries in children in Scotland, however, and what there is tends to focus on unintentional injuries where most progress has occurred. [4,5] Furthermore, although developmental stage is a recognised variable in injury risk [5-7], no recent exploration of the epidemiology of mortality by age in children in Scotland has been published.

The aim of this study was to contribute to the planning, implementation and evaluation of injury prevention measures in Scotland by providing a comprehensive description of mortality from injuries (intentional and unintentional) in children aged 0–14 years, both overall and by age-group.

## Methods

Anonymised data on all deaths in Scotland in children aged 0–14 years were obtained from the General Register Office for Scotland (GROS) for the period 2002–2006. Each record included data on age, sex, year and cause of death (coded using the Tenth Edition of the International Classification of Diseases (ICD10)). Mid-year population estimates obtained from GROS were used as the denominator in the calculation of mortality rates.

Data were grouped into six age categories (<1 year, 1–4 years, 5–9, 10–14, 0–14 and 1–14) and analysed in terms of number of deaths, mean annual mortality rates per 100,000 population, leading causes of death, and causes of injury death. Chi-square tests were used to determine statistical significance.

Ethical approval was not required as the data were anonymous and in the public domain.

## Results

### All injuries

In the period 2002–2006, 186 children aged 0–14 years died from an injury (includes poisoning) in Scotland. The overall injury mortality rate was 4.3 per 100,000 (Table 1). Injuries caused almost 1 in 10 deaths in children overall and 1 in 4 deaths in 1–14 year-olds. Moreover, they were the leading cause of death in the 5–9, 10–14 and 1–14 age-groups (Table 1). The proportion of all deaths caused by injury increased with increasing age. Tables 1 and 2 show that the injury mortality rate was highest in infants (age <1 year) and lowest in 5–9 year-olds; however, the biggest proportion of injury deaths occurred in the 10–14 age-group (40%) (Table 2).

**Table 1:** The most common causes of death per age-group, children aged 0–14 years, Scotland, 2002–06.

**Age-group (years)**	**Top five causes of death (ICD-10 code)**	**% of all deaths (MR per 100,000; n)**
**0 to 14**	Perinatal conditions (P00–96)	38% (17.4; 757)
	Congenital abnormalities (Q00–99)	19% (8.8; 385)
	**Injuries (V01–Y98)**	**9% (4.3; 186)**
	Malignant neoplasms (C00–97)	8% (3.6; 155)
	Sudden infant death syndrome (*R95)	8% (3.4; 150)
	*All causes of death*	*100% (45.9; 2002)*

**1–14**	**Injuries (V01–Y98)**	**25% (4.1; 167)**
	Malignant neoplasms (C00–97)	21% (3.4; 141)
	Congenital abnormalities (Q00–99)	14% (2.2; 91)
	Nervous system diseases (G00–99)	13% (2.1; 84)
	Respiratory system diseases (J00–99)	6% (1.1; 43)
	*All causes of death*	*100% (16.3; 669)*

**<1**	Perinatal conditions (P00–96)	56% (282.6; 752)
	Congenital abnormalities (Q00–99)	22% (110.5; 294)
	Sudden infant death syndrome (*R95)	11% (56.4; 150)
	Nervous system diseases (G00–99)	2% (8.6; 23)
	**Injuries (V01–Y98)**	**1% (7.1; 19)**
	*All causes of death*	*100% (501.0; 1333)*

**1 to 4**	Congenital abnormalities (Q00–99)	21% (5.6; 59)
	**Injuries (V01–Y98)**	**17% (4.4; 47)**
	Malignant neoplasms (C00–97)	16% (4.3; 46)
	Nervous system diseases (G00–99)	13% (3.4; 36)
	Infectious and parasitic diseases (A00–B99)	8% (2.2; 23)
	*All causes of death*	*100% (26.3; 280)*

**5 to 9**	**Injuries (V01–Y98)**	**29% (3.2; 46)**
	Malignant neoplasms (C00–97)	22% (2.4; 35)
	Nervous system diseases (G00–99)	14% (1.6; 23)
	Respiratory system diseases (J00–99)	8% (0.8; 12)
	Congenital abnormalities (Q00–99)	7% (0.8; 11)
	*All causes of death*	*100% (11.1; 160)*

**10 to 14**	**Injuries (V01–Y98)**	**32% (4.7; 76)**
	Malignant neoplasms (C00–97)	26% (3.8; 60)
	Nervous system diseases (G00–99)	11% (1.6; 25)
	Congenital abnormalities (Q00–99)	9% (1.3; 21)
	Endocrine, nutritional and metabolic diseases (E00–90)	7% (0.9; 15)
	*All causes of death*	*100% (14.5; 229****)***

Ordered by descending mortality rate and showing the percentage proportion of all deaths attributable to each cause, mortality rates per 100,000 and number of deaths, by age-group.

*R95: Sudden Infant Death Syndrome (SIDS) is a sub-classification of R00–99: Other/unknown causes of death. 88% of deaths (150/171) in R00–99 are attributed to SIDS. SIDS only applies to children <1 year but its relatively high mortality rate makes it the 5th most common cause of death in children aged 0–14 years.

**Table 2:** Injury deaths and mortality rates per 100,000, by age-group.

**Age (years)**	**Injury cause (ICD-10 code)**	**Proportion of injury deaths in age-group attributed to cause (n, MR) Proportion of cause in age-group**
**0–14**	Pedestrian road traffic injuries (V01–09)	19% (36, 0.83) 100%
	Non-pedestrian road traffic injuries (V10–29, V40–49, V70–79)	15% (28, 0.64) 100%
	Assault/homicide (including undetermined intent in <1 & 1–4 age-groups) X85-Y09 (Y10–34)	14% (26, 0.60) 100%
	Suffocation (including choking/asphyxiation) (W75–84)	12% (22, 0.50) 100%
	Fire (X00–09)	11% (21, 0.48) 100%
	Falls (W00–19)	7% (13, 0.30) 100%
	Drowning (W65–74)	6% (12, 0.28) 100%
	*Suicide (incl. undetermined intent in age 10–14) X60–84 (Y10–34)	6% (12, 0.28) 100%
	**Other unintentional injuries	5% (9, 0.21) 100%
	Exposure to inanimate mechanical forces (W20–49)	2% (4, 0.10) 100%
	Poisoning (X40–49)	2% (3, 0.07) 100%
	*All causes*	*100% (186, 4.27) 100%*
	*Unintentional*	*80% (148, 3.39) 100%*
	*Intentional (X60–84, X85-Y09, Y10–34)*	*20% (38, 0.87) 100%*

**1–14**	Pedestrian road traffic injuries	22% (36,0.88) 100%
	Non-pedestrian road traffic injuries	17% (28, 0.68) 100%
	Fire	12% (20, 0.49) 95%
	Suffocation (including choking/asphyxiation)	11% (18, 0.44) 82%
	Assault/homicide (incl. undetermined intent in 1–4 age-group)	10% (17, 0.41) 65%
	Suicide (incl. undetermined intent in age 10–14)	7% (12, 0.29) 100%
	Falls	7% (11, 0.27) 85%
	Drowning	6% (10, 0.24) 83%
	Other unintentional injuries	5% (8, 0.2) 89%
	Exposure to inanimate mechanical forces	2% (4, 0.1) 100%
	Poisoning	2% (3, 0.07) 100%
	*All causes*	*100% (167, 4.08) 90%*
	*Unintentional*	*83% (138, 3.37) 93%*
	*Intentional*	*17% (29, 0.71) 76%*

**<1**	Assault/homicide (incl. undetermined intent***)	47% (9, 3.38) 35%
	Suffocation (including choking/asphyxiation)	21% (4, 1.50) 18%
	Drowning	11% (2, 0.75) 17%
	Falls	11% (2, 0.75) 15%
	Fire	5% (1, 0.38) 5%
	Other unintentional injuries	5% (1, 0.38) 11%
	*All causes*	*100% (19, 7.14) 10%*
	*Unintentional*	*53% (10, 3.76) 7%*
	*Intentional*	47% (9, 3.38) 24%

**1–4**	Fire	28% (13, 1.22) 62%
	Assault/homicide (including undetermined intent****)	19% (9, 0.85) 35%
	Suffocation (including choking/asphyxiation)	19% (9, 0.85) 41%
	Pedestrian road traffic injuries	11% (5, 0.47) 14%
	Non-pedestrian road traffic injuries	9% (4, 0.38) 14%
	Drowning	4% (2, 0.19) 17%
	Falls	4% (2, 0.19) 15%
	Exposure to inanimate mechanical forces	2% (1, 0.09) 25%
	Other unintentional injuries	2% (1, 0.09) 11%
	Poisoning	2% (1, 0.09) 33%
	*All causes*	*100% (47, 4.42) 25%*
	*Unintentional*	*81% (38, 3.57) 26%*
	*Intentional*	*19% (9, 0.85) 24%*

**5–9**	Pedestrian road traffic injuries	30% (14, 0.97) 39%
	Non-pedestrian road traffic injuries	20% (9, 0.62) 32%
	Assault/homicide ***** (X85-Y09)	11% (5, 0.35) 23%
	Fire	11% (5, 0.35) 24%
	Drowning	7% (3, 0.21) 25%
	Falls	7% (3, 0.21) 23%
	Other unintentional injuries	7% (3, 0.21) 33%
	Suffocation (including choking/asphyxiation)	4% (2, 0.14) 9%
	Exposure to inanimate mechanical forces	2% (1, 0.07) 25%
	Poisoning	2% (1, 0.07) 33%
	*All causes*	*100% (46, 3.18) 25%*
	*Unintentional*	*89% (41, 2.83) 28%*
	*Intentional*	*11% (5, 0.35) 13%*

**10–14**	Pedestrian road traffic injuries	23% (17, 1.07) 47%
	Non-pedestrian road traffic injuries	20% (15, 0.95) 54%
	Suicide (including undetermined intent)	16% (12, 0.76) 100%
	Suffocation (including choking/asphyxiation)	9% (7, 0.44) 32%
	Falls	8% (6, 0.38) 46%
	Drowning	7% (5, 0.32) 42%
	Other unintentional injuries	5% (4, 0.26) 44%
	Assault/homicide (X85-Y09)	4% (3, 0.19) 14%
	Exposure to inanimate mechanical forces	3% (2, 0.13) 50%
	Fire	3% (2, 0.13) 10%
	Poisoning	1% (1, 0.06) 33%
	*All causes*	*100% (74, 4.67) 40%*
	*Unintentional*	*80% (59, 3.72) 40%*
	*Intentional*	*20% (15, 0.95) 39%*

Children aged 0–14 years, Scotland, 2002–2006.

Ordered by descending mortality rate. Includes proportions of all injuries attributable to cause by age-group and proportions of cause occurring in age-group.

* The 10–14 age-group was the only one with suicide (includes injuries of undetermined intent: n = 3, MR 0.19, 4% of injury deaths in 10–14 year-olds.) ** Other unintentional injuries comprises: Other land transport accidents, Water transport accidents, Exposure to animate mechanical forces, Exposure to electric current, radiation and extreme ambient air temperature and pressure, Exposure to forces of nature, Complications of medical and surgical care, Sequelae of external causes of morbidity and mortality. ICD-10 codes: V80–89, V90–94, W50–64, W85–99, X30–39, Y40–84, Y85–89 respectively (n = 2,1,1,1,2,1,1 (age 0–14)). *** n = 1, MR 0.38, 5% of injury deaths in infants. **** n = 3, MR 0.28, 6% of injury deaths in 1–4 year-olds. ***** There were no injuries of undetermined intent in the 5–9 age-group.

### Age-groups

Overall, there was a significant relationship between age-group and death from injury over the five-year period (p < 0.05). Furthermore, children aged 5–9 years were significantly less likely to die from an injury than those aged either 0–4 years (combined) or 10–14 years (p < 0.05). Of 0–4 year-olds, however, only infants were significantly more at risk compared with 5–9 year-olds (p < 0.01).

The proportion of all deaths per age-group attributable to injuries increased with increasing age, from 1% in infants to 32% in those aged 10–14 (Table 1).

### Infants (age <1 year)

Injuries were not a major cause of death in infants, causing only 1.4% of all deaths (n = 19) (Table 1). However, compared with the other three age-groups, they had the highest mortality rates for assault/homicide, injuries of undetermined intent, falls, drowning, suffocation and other unintentional injuries (Table 2). Mortality rates from intentional injuries were similar to those from unintentional injuries but assault/homicide was the leading cause of injury death and almost a quarter of children under 15 who died from an intentional injury was in this age-group (Table 2). The mortality rate for assault/homicide in infants was five times greater than that of 1–4 year-olds, nine times that of 5–9 year-olds and 16 times that of 10–14 year-olds.

Suffocation (including choking and asphyxiation) was the second leading cause of injury death in infants, accounting for about a fifth of injury fatalities. Drowning and falls each caused 11%. There was one death from each of fire and other causes, and one of undetermined intent, each contributing 5% to the total of injury deaths. However, no infant fatalities from poisoning or road-traffic injuries occurred in the study period.

### Age 1–4 years

Over the study period, 47 children aged 1–4 years died from injuries, accounting for 17% of all deaths in this age-group and making injuries the second leading cause of death. Twenty-five percent of all injury deaths in under-15s occurred in 1–4 year-olds (Tables 1 and 2). The leading cause of injury death was fire which accounted for 28% of injury fatalities (Table 2), and most fire-related deaths in children <15 years occurred in this age-group (62%). Suffocation was the second most common individual cause of injury death, comprising 19% of the total. Pedestrian and non-pedestrian road-traffic injuries caused 11% and 9% respectively of injury deaths.

The ratio of unintentional:intentional injuries was about 4:1. Nevertheless, assault/homicide was the third main cause of injury mortality in 1–4 year-olds, accounting for 13% of the total, and 27% of all deaths due to assault/homicide in children <15 years occurred in this age-group. Assault/homicide was the second equal leading cause of injury death when deaths from injuries of undetermined intent were included (Table 2).

Compared with other age-groups, 1–4 year-olds had the highest mortality rate for fire and the lowest for drowning and falls (Table 2).

### Age 5–9 years

5–9 year-olds were significantly less likely to die from an injury than children aged either 0–4 or 10–14 years (p < 0.05). Furthermore, they had the lowest all-cause mortality rate (Table 1) and the lowest mortality rates for intentional injuries and suffocation (Table 2). Nevertheless, 25% of all injury deaths in children <15 years occurred in 5–9 year-olds, equating to 46 lives lost and making injuries the leading cause of death in this age-group.

Road-traffic injuries were the leading causes of injury death, accounting for 50% of the total. There were, however, more pedestrian than non-pedestrian fatalities (14 vs. 9) and the proportion of injury deaths attributed to pedestrian casualties was greater in 5–9 year-olds than in any other age-group (Table 2).

The ratio of unintentional:intentional injuries was greatest in 5–9 year-olds (9:1). However, almost 25% of all assault/homicide deaths in children 0–14 years occurred in this age-group (23%), and – along with fire-related fatalities – this was the third leading cause of injury death.

### Age 10–14 years

There were 74 injury fatalities in 10–14 year-olds which comprised 40% of all injury deaths in children <15 years. Injuries were the leading cause of death in this age-group, causing almost a third of all deaths (Tables 1 and 2).

The leading causes of injury deaths were road-traffic injuries and the highest mortality rates for both pedestrian and non-pedestrian road-traffic injuries occurred in this age-group.

The ratio of unintentional:intentional injuries was 4:1. The 10–14 age-group was the only one with suicide. Of 15 intentional injury fatalities, 12 were attributed to suicide and injuries of undetermined intent and together these accounted for 16% of all injury deaths in this age-group and were the third leading cause of injury death (Table 2). The mortality rate from assault/homicide was lower in 10–14 year-olds than in all other age-groups (Table 2). Fire-related mortality was also lowest in this age-group.

### Injury causes

The leading cause of injury death in under-15s was pedestrian road-traffic injuries. This was followed by non-pedestrian road-traffic injuries, suffocation and assault/homicide (equal), fire, and falls. Together these six causes accounted for almost 80% of injury fatalities.

There were no infant deaths from either pedestrian or non-pedestrian road-traffic injuries but mortality rates for both increased with each successive age-group from 1–4 onwards. Half of all road-traffic injury fatalities occurred in the 10–14 age-group.

Suffocation was an important cause of injury death in all age-groups except 5–9 year-olds. The under-5s comprised the biggest proportion (59%) of fatalities due to this cause and suffocation was the second leading cause of injury death in infants and third in 1–4 year-olds. However, almost a third of suffocation cases occurred in the 10–14 age-group.

Twenty-one children died from fire-related injuries, most of whom were aged 1–4 years. The mortality rate from fire in 1–4 year-olds was at least three times that of infants and 5–9 year-olds, and more than nine times that of 10–14 year-olds.

Overall, falls accounted for only 7% of injury fatalities. Infants had the highest mortality rate although most fatalities were aged 10–14 years (46%). Drowning was also not a leading cause of injury death in under-15s, contributing only 6% of the injury total. Most victims were aged 10–14 years (42%) although infants had the highest mortality rate.

Poisoning and exposure to inanimate mechanical forces had the lowest mortality rates, each accounting for only 2% of all injury fatalities. There were no infant fatalities from either cause and mortality rates for both were similar in all other age-groups.

Thirty-eight children aged <15 years died from an intentional injury (MR 0.87) over the study period, i.e. from assault/homicide (n = 22, MR 0.50); suicide (n = 9, 0.21); and injuries of undetermined intent (n = 7, 0.16) (Table 2). The ratio of unintentional:intentional injuries was 4:1. Intentional injury mortality rates were highest in infants and lowest in 5–9 year-olds but the biggest proportion of intentional injuries (39%) occurred in the 10–14 year age-group and was largely attributable to suicide and injuries of undetermined intent.

The mortality rate from assault/homicide was highest in infants and lowest in 10–14 year-olds, decreasing with increasing age (Table 2). The relative importance of assault/homicide as a leading cause of injury death fell by age-group, from first in infants, second in 1–4 year-olds, third in 5–9 year-olds to eighth in 10–14 year-olds (Table 2). When all intentional injuries were considered together (i.e. assault/homicide, suicide and injuries of undetermined intent), they caused more deaths in under-15s than any single cause of unintentional injury.

## Discussion

These results show that, in Scotland, injuries remain an important cause of death in children (0–14 years) and that the overall level of risk and risk from individual causes vary considerably by age-group. Nonetheless, the average childhood injury mortality rate over the period 2002–06 was half of that reported 10 years previously [8], and the proportion of all deaths in children under-15 attributable to injuries (9%) signifies a welcome reduction following a long period during which it remained at around 14%. [8] These declines are likely to be a reflection of the notable reduction in mortality from unintentional injuries over recent years. [4]

Previous studies have reported that injuries were the leading cause of death in all children <15 years both in Scotland and the European Union. [8,9] A recent Finnish study, however, stated that injuries were the main cause of death in 1–14 year-olds but not in infants. [8-10] Our results were similar to the Finnish study; we found that injuries were the third leading cause of death overall (under-15s) but the main cause of death when infants were excluded. However, further analysis of our data revealed that, in Scotland, injuries were the leading cause of death in the two oldest age-groups only.

The extent of cause-specific variation by age-group revealed in this study is consistent with earlier reports. [11,12] Younger age-groups were at greatest risk from assault/homicide, suffocation and fire; older from road-traffic injuries. Injury prevention in children has largely targeted unintentional injuries, with impressive results. In comparison, intentional injuries have been relatively neglected and mortality rates have remained almost static for many years. [11] Our results reflected the consequences of this inequity; we showed that, collectively, intentional injuries now pose a bigger threat to the lives of under-15s in Scotland than any single cause of unintentional injury, and that infants are particularly at risk. Furthermore, it is possible that ours are conservative estimates as mortality data for intentional injuries can be subject to misclassification. [13]

The issue of suicide and its prevention in children is largely unexplored [14,15], possibly due to small numbers and difficulties in attributing deaths from undetermined intent. [16] Misclassification also means that it might be under- or over-estimated. [17,18] Scottish studies that did include children, however, have reported an increasing trend in suicide rates. [19] Nevertheless, we showed that in 2002–06, suicide was still not a major cause of injury death in children <15 years in Scotland. In the 10–14 age-group, however, its impact was considerable: it was the third leading cause of injury death accounting for more fatalities than each of falls, drowning, fire, suffocation, and assault/homicide. This suggests that it is essential to include this age-group in suicide prevention strategies.

Road-traffic injuries, particularly pedestrian, have been the main causes of injury death in under-15s for many years [8] and our study showed that, in Scotland, they remain so despite declining mortality rates. [20-22] Consistent with other UK studies, however, [12,21-23] we showed that this only applied to the oldest age-groups (5–9 and 10–14) and that younger children were more likely to die from other types of injury. Greater supervision of younger children and more appropriate use of child restraints in cars [24] may partly explain the age disparity. Recent legislation clarifying the use of child restraints [25] may therefore impact on future mortality rates for non-pedestrian road-traffic injuries.

Policies and legislation aimed at reducing the number of pedestrian casualties in Scotland have recently been implemented with the introduction of Home Zones, Safer Routes to School and 20 mph speed limits around schools. [22,26] However, a systematic review of injury prevention in children found little evidence addressing pedestrian injuries and suggested that research was biased towards causes and interventions that were relatively easy to investigate. [27] This raises the possibility that the persistent excess mortality from pedestrian injuries in older children found in our study may be partially attributable to a lack of information on effective prevention.

Our results indicate an excess in fire-related mortality in 1–4 year-olds, reflecting findings from other studies. [28,29] Most of this excess is likely to be attributable to fires started by playing with matches and lighters. [28,29] Fire-related injury prevention strategies have largely focused on domestic smoke alarm installation but, whilst being effective in preventing child deaths from other types of fire [27], these may not prevent mortality from those caused by fire-play. [28,29] This suggests that prevention initiatives should also focus on reducing the availability of matches and lighters to vulnerable children.

A limitation of this study is that the classification of causes of injury deaths in ICD-10 is inconsistent. While some categories are clearly defined causes of death, such as suffocation and poisoning; others describe a mix of circumstances, locations and mechanisms. For example, causes of death from transport injuries are described in terms of the location and mechanism, e.g. "Pedestrian injured in transport accident", "Car occupant injured in transport accident", and provide no information on the type of injuries sustained, e.g. head injuries. Despite their shortcomings, however, these data provide sufficient epidemiological information to inform preventive action.

In summary, this study has revealed significant variation by age-group in injury mortality rates in children and has highlighted the age-groups at most risk and the leading threats to each one. The only causes of injury death that did not exhibit age variation were poisoning, exposure to inanimate mechanical forces and those grouped into other unintentional injuries, all of which had very small numbers of fatalities. Despite the downward trend in injury mortality rates, our results suggest that there remains great potential for improvement. Over the study period, if the injury mortality rate in 0–14 year-olds had equalled that of the age-group with the lowest (5–9 year-olds), 47 children's lives would have been saved. Likewise, if the injury mortality rate in infants (the age-group with the highest) had equalled that of the lowest, 50% of injury deaths in infants would have been avoided.

## Conclusion

In Scotland, injuries remain an important cause of death in children but the extent of variation by age-group in overall level of risk and risk from individual causes suggest that interventions tailored more to age-group would maximize prevention. In particular, the threats from assault/homicide in infants, fire in 1–4 year-olds, pedestrian injury in 5–14 year-olds, and suicide in 10–14 year-olds need urgent attention.

## Abbreviations

MR: Mortality rate.

## Competing interests

The authors declare that they have no competing interests.

## Authors' contributions

DS devised the study. JP analysed the data and drafted the manuscript. DS edited and commented on the original draft. Both authors read and approved the final manuscript.

## Pre-publication history

The pre-publication history for this paper can be accessed here:


